# Editorial: Practical interventions to tackle burnout in healthcare staff

**DOI:** 10.3389/fpsyt.2024.1385206

**Published:** 2024-02-27

**Authors:** Sulmaz Ghahramani, Eka Chkonia

**Affiliations:** ^1^ Health Policy Research Center, Institute of Health, Shiraz University of Medical Sciences, Shiraz, Iran; ^2^ Department of Psychiatry, Tbilisi State Medical University, Tbilisi, Georgia

**Keywords:** intervention, burnout, healthcare staff, stress, organizational culture

Burnout (BO) is a three-dimensional affective response to ongoing work-related stress prevalent in settings where individuals spend more time helping others. It encompasses emotional exhaustion, depersonalization, and a sense of personal accomplishment loss (1, 2). This syndrome is common among healthcare practitioners, and research suggests it was considerably more widespread during the last pandemic, affecting half of them (3).

BO has detrimental repercussions for healthcare workers (4) and patients (5–7), emphasizing the importance of treating BO for individual healthcare professionals and the healthcare system.

The individual characteristics of healthcare workers and work-related issues such as excessive workload, long working hours, lack of control, and emotional pressures all contribute to this condition (8, 9). Organizational culture and leadership, including supportive management (Mengistie et al.), have also been shown to contribute to BO.

Addressing BO among healthcare workers is crucial to their well-being and the quality of patient treatment (10). Several review studies recommended strategies aiming at treating stress and BO in hospital settings, with varying degrees of success. These interventions include individual-focused methods (emotion regulation, self-care workshop, yoga, massage, mindfulness, meditation, stress management skills, and communication abilities training). Other interventions include structural or organizational methods (workload or schedule-rotation, stress management training program, group face-to-face delivery, teamwork/transitions, Balint training, debriefing sessions, and a focus group). Finally, there are combined interventions (Snoezelen, stress management and resiliency training, stress management workshop, and personal training to improve relationships with coworkers) (11–13).

These intervention studies demonstrated a promising clinical reduction in BO. BO is a complex problem that should be addressed using a packaged technique. It has been suggested that interventions are more successful when they are attentive to both individual-focused and structural or organizational strategies (12, 13). Here are some suggestions for these strategies.

Firstly, it is clear that, in addition to assessing the effectiveness of existing initiatives and identifying any gaps or areas for improvement, it is necessary to determine which interventions are most effective in specific populations and how individual and organizational solutions can be combined to deliver even greater improvements in wellbeing than those achieved with individual solutions (Adam et al.).

Secondly, among the various strategies for reducing BO in healthcare providers, it is critical to establish and implement “practical” initiatives. For an intervention to be practical, it should be easily accessible in the workplace, healthcare workers should be aware of it, it should address their unmet counseling and support needs (Diehl et al.), and its justification should align with the healthcare provider’s workload. For this purpose, novel interventions given via digital technology (Adam et al.), such as virtual reality, appear to be a potential alternative for reducing the negative effects of stress and BO.

Thirdly, the importance of leadership in building a healthy and supportive work environment should not be neglected. Recent research has attempted to explore the impact of organizational responsibility in influencing employee outcomes, known as “corporate social responsibility (CSR)”. CSR aims to deliver context-specific solutions for the benefit of all stakeholders in terms of the triple bottom line effect, which incorporates economic, social, and environmental responsibilities in an organization (14). It has been demonstrated that micro-elements of CSR negatively predict BO, albeit this link is controlled by work engagement and intrinsic drive and modulated by compassion at work (Chen and Liu). However, we recommend conducting more context-specific research to validate the findings in other healthcare workers and geographic regions.

Finally, measurements and assessment tools should be used to assess the effectiveness of practical interventions. Strategies must be continuously evaluated and adapted in response to input and outcomes.

We propose a collaborative effort among healthcare professionals, leaders, politicians, and academics to comprehensively address practical interventions for reducing BO.

## Author contributions

SG: Writing – original draft, Writing – review & editing. EC: Writing – review & editing.
